# CELF4 (rs1786814) gene polymorphism and speckle-tracking Echocardiography for cardiovascular complications in childhood cancer survivors

**DOI:** 10.1038/s41390-024-03400-3

**Published:** 2024-07-24

**Authors:** Seham M. Ragab, Mahmoud A. El-Hawy, Sally M. El-Hefnawy, Hend M. A. El –Deeb, Amany S. Elfalah, Asmaa A. Mahmoud

**Affiliations:** 1 Departement of Pediatrics, Faculty of Medicine, Menoufia University, Shebin ElKom, Egypt; 2 Medical Biochemistry and Molecular Biology Departement, Faculty of Medicine, Menoufia University, Shebin Elkom, Egypt; 3 Medical Biochemistry Department, Faculty of Dentistry, AlRyada University for Science and Technology (RST), Al-Sadat City, Egypt; 4 Department of Cardiology, Faculty of Medicine, Menoufia University, Shebin Elkom, Egypt

## Abstract

**Background:**

Despite a well-known dose-dependent association between the risk of cardiac dysfunction and anthracycline, the risk of cardiac dysfunction for any given anthracycline dose varies between patients. So, we assessed CELF4 (rs1786814) gene polymorphism on anthracycline-related cardiotoxicity in childhood cancer survivors (CCS).

**Methods:**

This comparative cross-sectional study included 53 CCS who had regular follow-up visits at the Pediatric Oncology Unit, Menoufia University Hospital. CELF4 (rs1786814) gene polymorphism and conventional and speckle-tracking Echocardiography were done for all survivors.

**Results:**

Regarding CELF4 (rs1786814) genotypes, significant differences existed between the studied groups with a predominance of GG homozygous mutation. For Echocardiographic findings, the ejection fraction and end-systolic diameter compared to the control group, were significantly lower in the survivors group. Speckle- tracking Echocardiography showed a significant difference regarding (GLPS-A4C) and (GLPS-LAX), with no significant difference regarding (GLPS-A2C), (GLPS-Avg) and left atrium between the studied groups. Multivariate logistic regression analysis illustrated a statistically significant relation between cumulative anthracycline dose >300 mg/m^2^ and CLEF4 (rs1786814) genotypes (GG and GA) and the risk of cardiotoxicity with more significance in GG mutation.

**Conclusion:**

Early detection of ventricular dysfunction in CCS with subclinical cardiotoxicity with regular follow-up is promising before the development of life-threatening complications.

**Impact:**

- Early detection of anthracycline-related cardiotoxicity in childhood cancer survivors (CCS) after finishing chemotherapy.
- CLEF4 (rs1786814) GG variant is more significant in CCS exposed to high-dose anthracycline.
- GLPS holds promise as an early predictor of late left ventricular dysfunction and subclinical cardiotoxicity in CCS.

## Introduction

Cardiovascular dysfunction caused by chemotherapy is a serious side effect of cancer treatment.^1^ Survivors of childhood cancer are more prone to death from cardiac problems. This increased risk of cardiac death is due to exposure to cardiotoxic treatment such as anthracycline.^2^

CELF4 is an RNA binding protein mediating the alternative splicing of the TNNT2 gene, that encodes cardiac troponin T.^3^ CELF4 variants with reduced activity to target exon 5 inclusion of TNNT2 pre-mRNA enable the continued expression of embryonic cardiac troponin T in the adult heart helping thin myofilaments to handle increasing calcium concentrations which causes decreased ventricular pumping efficiency.^4^ The SNP rs1786814 in CELF4 is associated with anthracycline-induced cardiomyopathy in CCS.^5^

The subendocardial layer of the ventricle was particularly sensitive to doxorubicin damage. Speckle tracking echocardiography allowed non-invasive evaluation of early systolic dysfunction.^6^

Therefore, this research goal was to detect the modifying effect of CELF4 (rs1786814) gene polymorphism on anthracycline-related cardiotoxicity in survivors of childhood cancer.

## Subjects and methods

### Study design

A comparative cross-sectional study was done on 53 CCS who were presented with acute lymphoblastic leukemia and lymphoma who finished chemotherapy treatment. They were gathered from the Pediatric Oncology Unit, Menoufia University Hospital during their follow-up visits from May 2021 to May 2022.

Fifty-three healthy children with matched age, sex, and socioeconomic standards were recruited as a control group. Presence of any congenital or acquired cardiac disease before their diagnosis with cancer was omitted from the study.

The Faculty of Medicine’s Ethical Committee at Menoufia University gave its approval to the study. Approval ID number (5/2020 PEDI 28).

### Study methods

Full history taking, thorough clinical examination, anthropometric measurements, cardiac imaging assessment by conventional 2D echocardiography and 2D speckle- tracking echocardiography, molecular genetic study, and genotyping of the CELF4 (rs1786814) gene polymorphism by allelic discrimination assay using real-time PCR technique were done for all studied groups.

#### Sample collection and preparation

2 ml of venous blood was collected and then transferred into an EDTA tube for DNA extraction and PCR.

#### DNA extraction

A Thermo Scientific GeneJET Genomic DNA purification kit from Lithuania was used to extract DNA from whole blood. DNA was extracted and kept at −200 C for subsequent PCR steps. Proteinase K is used in the provided Lysis Solution to digest the samples. Once the lysate has been loaded onto the purification column and combined with ethanol, the DNA begins to bind to the silica membrane. By using the ready-made Wash Buffers to wash the column, impurities are successfully eliminated. The Elution Buffer is then used to elute genomic DNA under low ionic strength conditions.

#### Genotyping of CELF4 gene (rs1786814) by real-time PCR

The CELF4 gene’s rs1786814 polymorphism was genotyped using real-time PCR and an allelic discrimination assay with a TaqMan probe from Applied Biosystems in the United States. Thermo Scientific also supplied the Master Mix (40X), primers, and probes. Sequences of the following probes were used: [VIC/FAM] GCAAGGCCGGTTTCATGCAAAACAA [A/G]CTCTGAGCATGGAAAGGATTTAAGC. A mixture of 10 µl of Master Mix and 3.5 µl of nuclease-free water was added to 1.5 µl of the primer/probe mixture. For each reaction, five microliters of our extracted DNA were added. The cycling parameters were set to include a preliminary denaturation phase lasting 10 min at 95 °C, 40 cycles consisting of 15 s of denaturation at 94 °C, 60 s of primer annealing at 50 °C, and 2 min of extension at 72 °C, and finally, a terminal extension phase lasting one minute at 72 °C. Data were examined using V.2.0.1 of the software that came with the ABI7500 real-time PCR instrument **(**Supplementary 1, 2).

#### Study imaging

##### Echocardiography

Standard transducer positions were used to perform echocardiographic imaging in the left lateral decubitus, parasternal long, short-axis, apical 2 and 4-chamber views. Utilized was GE Vivid 9 Norton Norway with a multi-frequency 1.7–4 MHz MS5 transducer. LA diameter and volume were measured, along with LV end-diastolic diameter (EDD), end-systolic diameter (ESD), ejection fraction (EF%), and other parameters.

##### Speckle-tracking echocardiography

Two-dimensional loop views from four-chamber, three-chamber, and two-chamber views were obtained for the measurement of LV longitudinal strain. For off-line analysis, all recordings that included at least three cardiac cycles were digitally stored. The machine software version 113, which automatically displays the end-systolic frame of the cardiac cycle, opened stored images. The endocardial border is manually drawn in the end-systolic frame, starting at one end of the mitral annulus and ending at the other. After that, the software creates an ROI that contains the entire myocardial thickness.

The software creates segmental and overall longitudinal strain by segmenting the LV myocardium into six sections. The global longitudinal strain (GLS), which was calculated by averaging the strain values for each segment, was measured.

Additionally, the ultrasound system offers a Bull’s-eye representation of the local and global longitudinal strain.^7^ With the help of a (Vivid 7 Pro, 7 MHz, and 3 MHz transducer, GE, Horten, Norway) detailed echocardiography was carried out.

### Sample size determination

Considering previous research (Wang et al.) ^5^ who studied the CELF4 gene-environment (anthracycline) interaction effect and reported that percentages of <300 mg/m^2^ and GG in cases and control groups were 39% and 15% respectively. Minimum appropriate calculated sample size (which achieves the power of ≥ 80% and equals 0.84) was calculated as 106 participants.

### Statistical analysis

With the help of SPSS (version 20, SPSS Inc, Chicago, IL), data were statistically analyzed. The Chi-square (2) test, Student’s *t*-test, and Mann-Whitney test were among the analytical statistics used. When comparing three or more groups with normally distributed quantitative variables, ANOVA (f) is used. To assess the relationship between two quantitative variables, Spearman correlation (r) was used. Calculating the effects of various risk factors as independent odds ratios required the use of multivariate logistic regression and binary logistic regression. P-value less than 0.05% was regarded as statistically significant.

## Results

### The basic characteristics of the studied groups are shown in Table 1

A total of 53 CCS patients and 53 controls were included. The weight and BMI were significantly elevated in the survivors than in the controls

**Table 1:** Demographic characteristics and anthropometric measurements among studied groups.

Parameters	Survivors Group (No. = 53)	Control Group (No. = 53)	Test of significance	*P* value
**Age (years)**				
Median (Range)	11 (5.5–18)	11 (5–18)	Mann Whitney test = 1.26	0.21
**Sex**				
Male	32 (60.4%)	28 (52.8%)		
Female	21 (39.6%)	25 (47.2%)	χ^2^ = 0.61	0.43
**Weight (Kg)**				
Median (Range)	41 (19.9–103)	36 (17–76)	Mann Whitney test = 5.37	0.03^a^
**Height (cm)**				
Mean ± SD	144.4 ± 18.5	137.1 ± 20.9	*t*-test = 1.90	0.06
**BMI (kg/m**^**2**^**%)**				
Mean ± SD	21.4 ± 5.4	19.1 ± 3.8	*t*-test=2.55	0.01^a^

^a^significant difference; *t*-test: student *t* test; χ^2^ test Chi square test, *BMI* body mass index.

### CELF4 (rs1786814) gene polymorphism and speckle-tracking Echocardiographic findings of the left ventricle are shown in Table 2

Regarding the genotyping of CELF4 (rs1786814) genotypes, there was a significant variation between survivors and controls. It was found that CELF4 (rs1786814) genotype GG was more significant than GA and AA mutations (*P* < 0.001).

**Table 2:** Comparison between studied groups regarding CELF4 (rs1786814) gene polymorphism and speckle-tracking Echocardiographic findings of left ventricle.

Parameters	Survivors group (No.= 53)	Control group (No.= 53)	χ^2^ test (*P* value)	OR (95% CI)
rs1786814 genotypes				
AA	9 17.0%	29 54.7%	–	1
GA	14 26.4%	16 30.2%	23.23 (*p* < 0.001**)	2.82 (1.00–7.95)
GG	30 56.6%	8 15.1%		12.08 (4.10–35.6)
**rs1786814**				
AA and GA	23 43.4%	45 84.9%	–	1
GG	30 56.6%	8 15.1%	19.85 (*P* < 0.001**)	7.34 (2.9-18.55)
**rs1786814 alleles**				
A	32 30.2%	74 69.8%	–	1
G	74 69.8%	32 30.2%	33.28 (*P* < 0.001**)	5.35 (2.97–9.61)
**Parameters of speckle-tracking Echocardiographic findings**	**Survivors group (No.= 53)**	**Control group (No.= 53)**	***t*–test**	***P*** **value**
**GLPS -A4 (%)**				
**Mean** **±** **SD**	21.3 ± 3.2	22.3 ± 1.7	2.05	0.04^a^
**GLPS-LAX (%)**				
**Mean** **±** **SD**	20.9 ± 3.7	22.0 ± 1.4	2.06	0.04^a^
**GLPS-A2 (%)**				
**Mean** **±** **SD**	22.0 ± 2.9	22.2 ± 1.4	0.33	0.75
**GLPS-AVG (%)**				
**Mean** **±** **SD**	21.3 ± 2.7	22.0 ± 0.94	1.90	0.06

^a^significant difference **highly significant difference; *χ2 test* Chi square test; *t* student’s *t* test; *OR* Odds Ratio; *CI* Confidence Interval; GA (heterozygous mutant); GG (homozygous mutant); AA (homozygous wild type); *GLPS* global longitudinal peak systolic strain; *LAX* apical long axis view; *A4C* apical four-chamber view; *A2C* apical two-chamber view; *Avg* average.

Regarding Speckle-tracking Echocardiography (STE) findings, analysis of the left ventricle global longitudinal peak systolic strain (GLPS) of the three apical views, the GLPS of apical four-chamber view (GLPS-A4C), the GLPS of apical two-chamber view (GLPS-A2C), the GLPS of apical long axis view (GLPS-LAX), and the average GLPS (GLPS-Avg), there was a significant difference in (GLPS-A4C) and (GLPS-LAX) between the studied groups (*P* = 0.04).

### The relationship between CELF4 (rs 1786814) gene polymorphism and conventional and speckle-tracking Echocardiographic findings among the survivors are shown in Table 3

Survivors with GG homozygous mutation versus those with AA homozygous wild mutation showed a significantly low mean EDD (*P* = 0.007) in comparison to survivors with GA heterozygous mutation versus those with AA mutation and survivors with GG homozygous mutation versus those with GA heterozygous mutation. Survivors with GG homozygous mutation versus those with GA heterozygous mutation showed a significantly low mean ESD (*P* = 0.009) in comparison to survivors with GG homozygous mutation versus those with AA homozygous wild mutation and survivors with GA heterozygous mutation versus those with AA homozygous wild mutation.

**Table 3:** Relationship between CELF4 (rs 1786814) gene polymorphism and conventional and speckle-tracking Echocardiographic findings among survivors group.

Parameters	Survivors group			ANOVA test and *P* value	Post hoc test
	AA (No.= 9)	GA (No.= 14)	GG (No.= 30)		
**GLPS-A4 (%)**					P1 = 0.07
**Mean** **±** **SD**	19.1 ± 2.6	21.5 ± 3.1	21.9 ± 3.3	Test = 2.93	P2 = 0.02^a^
				*P* = 0.06	P3 = 0.68
**GLPS-LAX (%)**					P1 = 0.19
**Mean** **±** **SD**	18.9 ± 3.4	21.0 ± 5.0	21.5 ± 2.8	Test = 1.68	P2 = 0.07
				*P* = 0.19	P3 = 0.71
**GLPS-A2 (%)**					P1 = 0.44
**Mean** **±** **SD**	20.4 ± 1.7	21.3 ± 4.1	22.8 ± 2.4	Test = 3.14	P2 = 0.03^a^
				*P* = 0.05	P3 = 0.11
**GLPS-AVG (%)**					P1 = 0.16
**Mean** **±** **SD**	19.5 ± 1.6	21.1 ± 3.6	21.9 ± 2.4	Test = 2.95	P2 = 0.02^a^
				*P* = 0.06	P3 = 0.34
					P1 = 0.19
**EDD (Cm)**	4.6 ± 0.69	4.4 ± 0.42	4.1 ± 0.33	Test = 4.34	P2 = 0.007^a^
**Mean** **±** **SD**				*P* = 0.01^a^	P3 = 0.12
					P1 = 0.65
**ESD(Cm)**	2.9 ± 0.55	2.9 ± 0.31	2.6 ± 0.18	Test = 6.03	P2 = 0.007^a^
**Mean** **±** **SD**				*P* = 0.005^a^	P3 = 0.009^a^
					P1 = 0.88
**EF (%)**	65.2 ± 4.2	65.4 ± 2.8	66.4 ± 2.7	Test = 0.82	P2 = 0.31
**Mean** **±** **SD**				*P* = 0.45	P3 = 0.32
					P1 = 0.86
**FS (%)**	35.3 ± 2.9	35.1 ± 2.4	35.6 ± 2.4	Test = 0.17	P2 = 0.78
**Mean** **±** **SD**				*P* = 0.85	P3 = 0.58

^a^significant difference; *GLPS* global longitudinal peak systolic strain, *LAX* apical long axis view, *A4C* apical four-chamber view, *A2C* apical two-chamber view, *Avg* average, *EF* Ejection Fraction, *FS* Factional Shortening, *EDD* end-diastolic diameter, *ESD* end- systolic diameter, GA (heterozygous mutant) and GG (homozygous mutant), AA (homozygous wild type), P1: survivors with AA versus those with GA; P2: survivors with AA versus those with GG P3: survivors with GA versus those with GG.

Survivors with GG homozygous mutation versus those with AA homozygous wild mutation showed low mean GLPS-A4 (%), GLPS-LAX (%) and GLPS-A2 (%) (*P* = 0.02) in comparison to survivors with GG homozygous mutation versus those with GA heterozygous mutation and survivors with GA heterozygous mutation versus those with AA homozygous wild mutation.

### Comparison between survivor subgroups by cumulative dose of Doxorubicin (mg/m^2^) regarding conventional and speckle-tracking Echocardiographic findings are shown in Table 4

A statistically significant difference was found in EF %, FS %, and GLPS-LAX (%) (*P* = 0.004; 0.01; and 0.01 respectively) between survivor subgroups regarding the cumulative dose of Doxorubicin (mg/m^2^): ( ≤ 200 mg/m^2^), and ( > 200 mg/m^2^).

**Table 4:** Comparison between survivor subgroups by cumulative dose of Doxorubicin (mg/m^2^) regarding conventional and speckle-tracking Echocardiographic findings.

Parameters	Cumulative dose of Doxorubicin (mg/m^2^) in survivors group		*t*–test	*P* value
	≤200 (No. = 28)	>200 (No. = 25)		
	Mean ± SD	Mean ± SD		
**EDD (Cm)**	4.3 ± 0.38	4.3 ± 0.54	0.61	0.54
**ESD (Cm)**	2.7 ± 0.24	2.8 ± 0.41	1.31	0.19
**EF (%)**	67.1 ± 2.2	64.7 ± 3.4	3.03	0.004^a^
**FS (%)**	36.2 ± 2.3	34.6 ± 2.4	2.55	0.01^a^
**GLPS-A4 (%)**	21.3 ± 3.8	21.3 ± 2.5	0.01	0.99
**GLPS-LAX (%)**	19.8 ± 3.6	22.2 ± 3.4	2.56	0.01^a^
**GLPS-A2 (%)**	22.3 ± 3.2	21.7 ± 2.8	0.72	0.47
**GLPS-AVG (%)**	21.0 ± 2.9	21.5 ± 2.5	0.73	0.47
**LA (Cm)**	3.1 ± 0.35	3.1 ± 0.34	0.76	0.45

^a^significant difference; *GLPS* global longitudinal peak systolic strain, *LAX* apical long axis view, *A4C* apical four-chamber view, *A2C* apical two-chamber view, *Avg* average, *LA* left atrium, *EF* Ejection Fraction, *FS* Factional Shortening, *EDD* end-diastolic diameter, *ESD* end-systolic diameter, *LA* left atrium.

### Multivariate logistic regression analysis for the prediction of the risk of cardiotoxicity among survivors is shown in Table 5

There was a statistically significant relation between height, BMI, and CLEF4 rs1786814 genotype and the risk of cardiotoxicity (*P* = 0.03) with a highly significant difference in cumulative anthracycline dose >300 mg/m^2^ and GG genotype and the risk of cardiotoxicity (*P* < 0.001).

**Table 5:** Multivariate logistic regression analysis for the prediction of risk of cardiotoxicity among survivors.

Variable	Adjusted OR	95% CI	*p*-value
**Age (years)**	0.96	0.76–1.23	0.77
**Sex**			
Male	1	1	–
Female	1.84	0.68–4.93	0.23
**Weight (Kg)**	0.82	0.67–1.00	0.06
**Height (Cm)**	1.13	1.01–1.27	0.03^a^
**BMI (kg/m**^**2**^**%)**	1.70	1.04–2.78	0.03^a^
**Cumulative dose of Doxorubicin** > **300** **mg/m**^**2**^	4.65	2.52–10.81	<0.001**
**rs1786814 genotypes**			
AA	1	1	–
GA	3.26	1.06–10.04	0.03^a^
GG	9.05	2.87–28.57	<0.001**

^a^significant difference **highly significant difference; *OR* Odds Ratio, *CI* Confidence Interval, *BMI* body mass index.

### Correlation between speckle-tracking Echocardiographic (STE) findings of LV and other parameters are shown in Figs. 1, 2

A statistically significant positive correlation was reported between GLPS-LAX and cumulative dose of Doxorubicin (mg/m^2^) (*r* = 0.308; *P* = 0.03); and a statistically significant negative correlation with FS% (*r* = −0.305; *P* = 0.03) and LA (*r* = −0.374; *P* = 0.006). There was a statistically significant positive correlation between GLPS-A2 and EF% (*r* = 0.288);

**Fig. 1:**
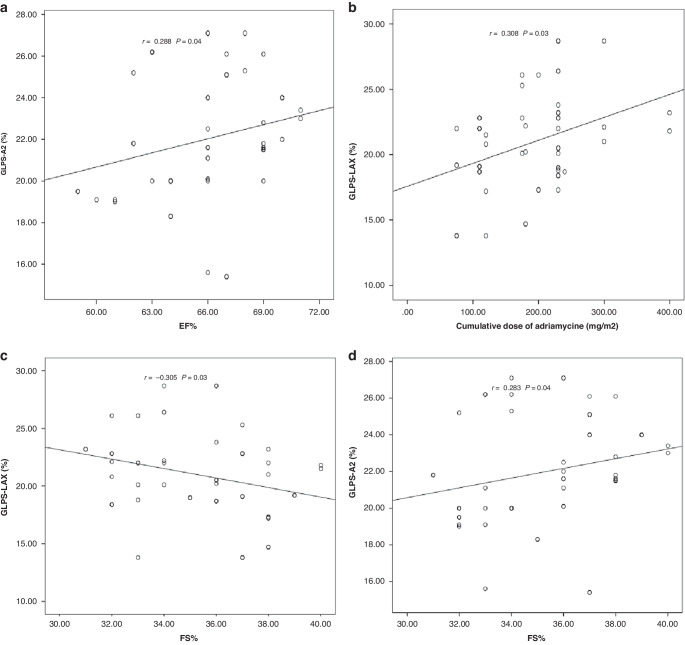
Correlation between speckle-tracking Echocardiographic findings of left ventricle and other parameters. **A** Positive correlation between GLPS-A2 and EF%. **B** Positive correlation between GLPS-LAX and cumulative dose of adriamycin (mg/m^2^). **C** Negative correlation between GLPS-LAX and FS%. **D** Positive correlation between GLPS-A2 and FS%.

**Fig. 2:**
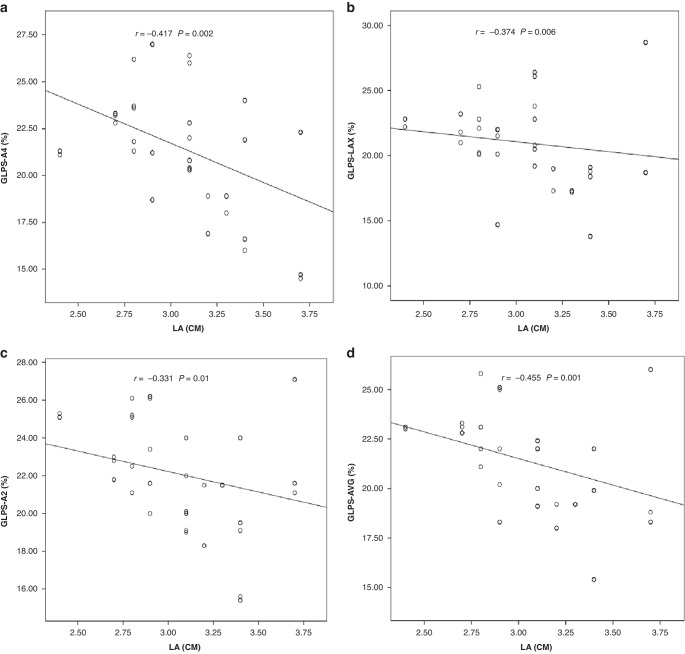
Correlation between speckle-tracking Echocardiographic findings and left atrium. **A** Negative correlation between GLPS-A4 and left atrium. **B** Negative correlation between GLPS-LAX and left atrium. **C** Negative correlation between GLPS-A2 and left atrium. **D** Negative correlation between GLPS-AVG and left atrium.

*(P* = 0.04), and FS% (*r* = 0.283; *P* = 0.04) and a statistically significant negative correlation with LA (*r* = −0.331; *P* = 0.01). There was a statistically significant negative correlation between GLPS-AVG and LA (*r* = −0.455; *P* = 0.001); and between GLPS-A4 (%) and LA (*r* = −0.417; *P* = 0.002).

## Discussion

Long-term CCS are more likely to develop heart disease and die from heart-related causes.^8^ Cardiologists and oncologists must now detect cardiotoxicity early to stop irreversible myocardial damage and heart failure caused by chemotherapy.^9^

Regarding the genotyping of CELF4 (rs1786814) genotypes, it was found that CELF4 (rs1786814) genotype GG was more significant than GA and AA mutations.

Regarding Speckle Tracking Echocardiography findings, there was a significant variation in (GLPS-AVG), (GLPS-A4C) and (GLPS-LAX) between the studied groups.

Previous studies^1,5^ found that the CLEF4 (rs1786814) GG variant was more significant in survivors than in controls. *Wang* et al.^5^ reported that the GG genotype was linked with a high risk of anthracycline-induced cardiomyopathy compared to the GA/AA genotype.

El Rashedi et al.^10^ showed that the average GLPS was significantly lower in the survivors than in controls. Yu et al.^11^ found lower mean FS, LVEF, and GLPS (LV systolic dysfunction) in survivors than in controls. Abnormal GLPS was illustrated in 18% of patients.

Pignatelli et al.^12^ found that 60% of survivors showed abnormal GLPS findings and Armstrong et al.^13^ found that 5.8% of the survivors had LVEF < 50%. However, LV systolic dysfunction distinguished by global longitudinal and global circumferential strain was more predominant than an abnormal LVEF.

Thavendiranathan et al.^14^ reported that an early reduction of 10% to 15% in GLPS (asymptomatic and symptomatic LV systolic dysfunction) was considered the best predictor of late cardiotoxicity in survivors receiving cardiotoxic drugs.

This study showed a statistically significant difference in EF%, FS%, and GLPS-LAX% between survivor subgroups regarding the cumulative dose of Doxorubicin being lower in those with doses >200 mg/m^2^.

Al-Biltagi et al.^15^ found that the average GLPS of the LV showed a significant reduction in the patient group prior to and after the doxorubicin treatment. *Armstrong* et al.^13^ found that abnormal GLPS was associated with anthracycline dose >300 mg/m^2^.

Park et al.^16^ illustrated that there was global longitudinal dysfunction with a low anthracycline dose of <300 mg/m^2^.

Longitudinal strain values are reduced throughout anthracycline treatment and at the latest follow-up ≥ 5 years after anthracycline exposure. ^17^

Multivariate logistic regression analysis for the prediction of the risk of cardiotoxicity among survivors showed a statistically significant relation between height, BMI, cumulative anthracycline dose >300 mg/m^2^ and CLEF4 (rs1786814) genotype and the risk of cardiotoxicity with a highly significant difference in anthracycline dose and GG genotype and the risk of cardiotoxicity.

*Bhati*.^18^ illustrated multivariable analysis adjusted for anthracycline dose revealed that among patients who received >300 mg/m^2^ of anthracycline, compared to people with GA/AA genotypes and anthracycline exposure of 300 mg/m^2^ or less, those with the rs1786814 GG genotype had a 10.2-fold higher risk of cardiomyopathy.

According to Wang et al.^5^ ‘s multivariable conditional logistic regression analysis with anthracycline dose adjustment, the GG genotype was linked to a higher risk of cardiomyopathy than the GA/AA genotype. Patients with the GG genotype who were exposed to high doses of anthracycline ( > 300 mg/m^2^) had a 10.16-fold higher risk of developing cardiomyopathy than those with the GA/AA genotype who were exposed to low-to-moderate doses (300 mg/m^2^).

The cumulative dose of doxorubicin and GLPS-LAX showed a statistically significant positive correlation, while FS% and LA showed a statistically significant negative correlation. Between GLPS-A2, EF%, and FS% there was a statistically significant positive correlation, and there was a statistically significant negative correlation with LA. Both GLPS-A4 (%) and LA showed a statistically significant negative correlation between them.

Mavinkurve-Groothuis et al. ^19^ and Cheung et al. ^20^ found that the cumulative anthracycline dose correlated negatively with global longitudinal strain.

*Yu* et al. ^11^ found that there was no significant correlation between echocardiographic parameters of LV systolic or diastolic function (by 2D echocardiography or 2D STE) and cumulative anthracycline dosage. *Pignatelli* et al. ^12^ showed no significant relationship between GLPS and the cumulative dosage of anthracycline. *Poterucha* et al.^21^ found that LVEF, and cumulative anthracyclines dosage were not associated with any other strain parameter. *Mavinkurve-Groothuis* et al.^22^ found that there was no significant correlation between GLPS and cumulative anthracycline dose.

The production of free radicals and superoxides is the mechanism by which anthracycline cause cardiotoxicity. Cardiomyocyte loss or damage brought on by anthracycline reduces the quantity of remaining myocardial cells needed to produce a typical myocardial mass.^9^ In the first six years following anthracycline therapy, cardiomyocyte loss causes LV wall thinning and progressive LV dilation.^23^ So, Early diagnosis of ventricular dysfunction in asymptomatic cardiac subjects before the development of life-threatening complications is recommended.

## Conclusion

CLEF4 (rs1786814) GG variant is more significant in patients exposed to high-dose anthracycline. GLPS holds promise as an early predictor of late left ventricular dysfunction and subclinical cardiotoxicity.

## Limitation of our study

A small sample size and loss of tracking can make it difficult to acquire at the right frame rate.

## Supplementary information

Supplementary information

Supplementary information

## Data Availability

The corresponding author will provide the datasets produced and/or analyzed during the current study upon reasonable request.
