# Supplementary figures and images for: CELF4 (rs1786814) gene polymorphism and speckle-tracking Echocardiography for cardiovascular complications in childhood cancer survivors
Source: Pediatr Res. 2024 Jul 24;98(3):989–96. doi: 10.1038/s41390-024-03400-3 (PMC12507695; doi:10.1038/s41390-024-03400-3)

**Allelic Discrimination Plot**

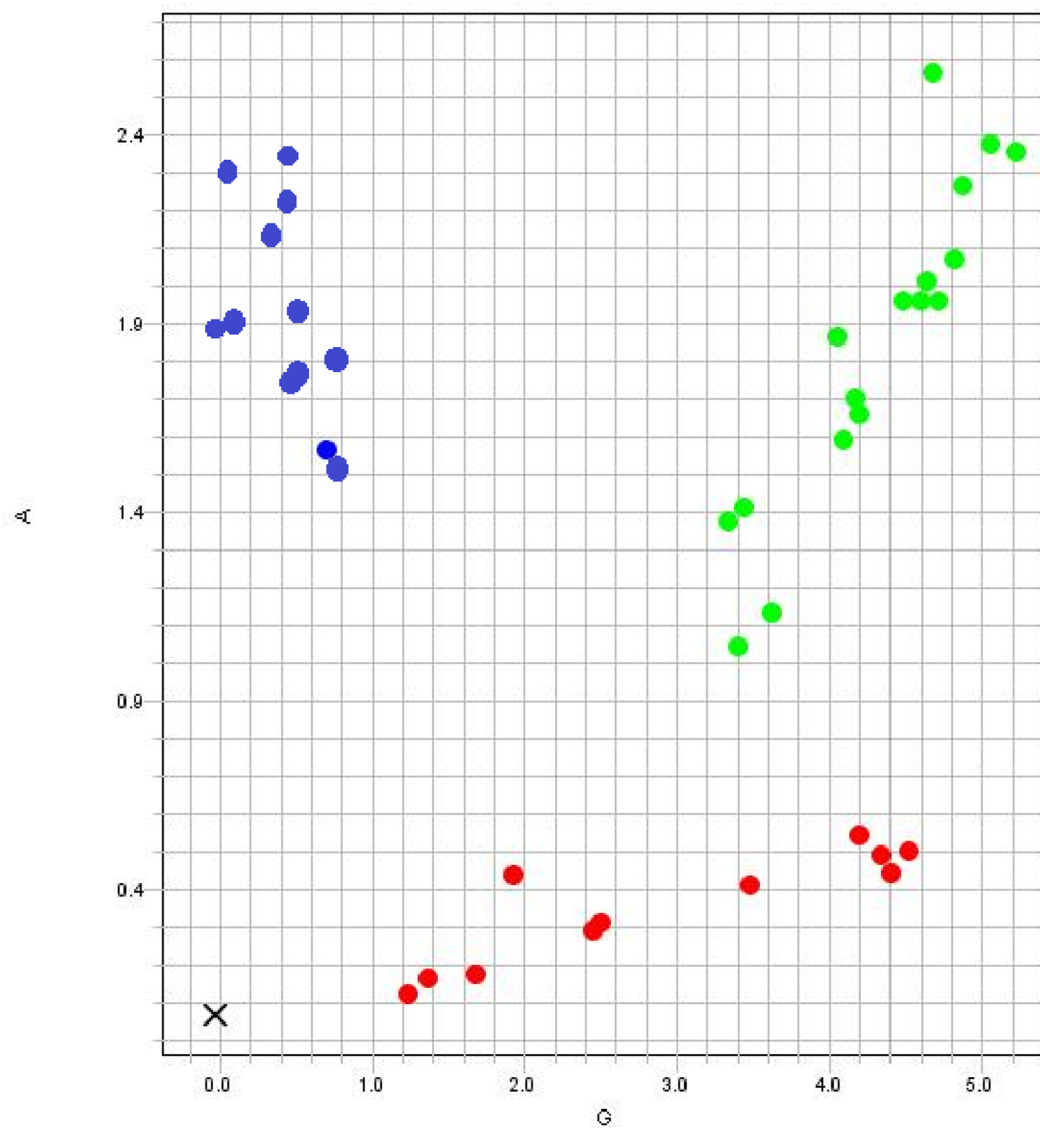

**Legend**

● G / G

● G / A

● A / A

X Undetermined

Supplement: Supplementary file 1 — Supplementary information [file 41390_2024_3400_MOESM1_ESM.pdf]

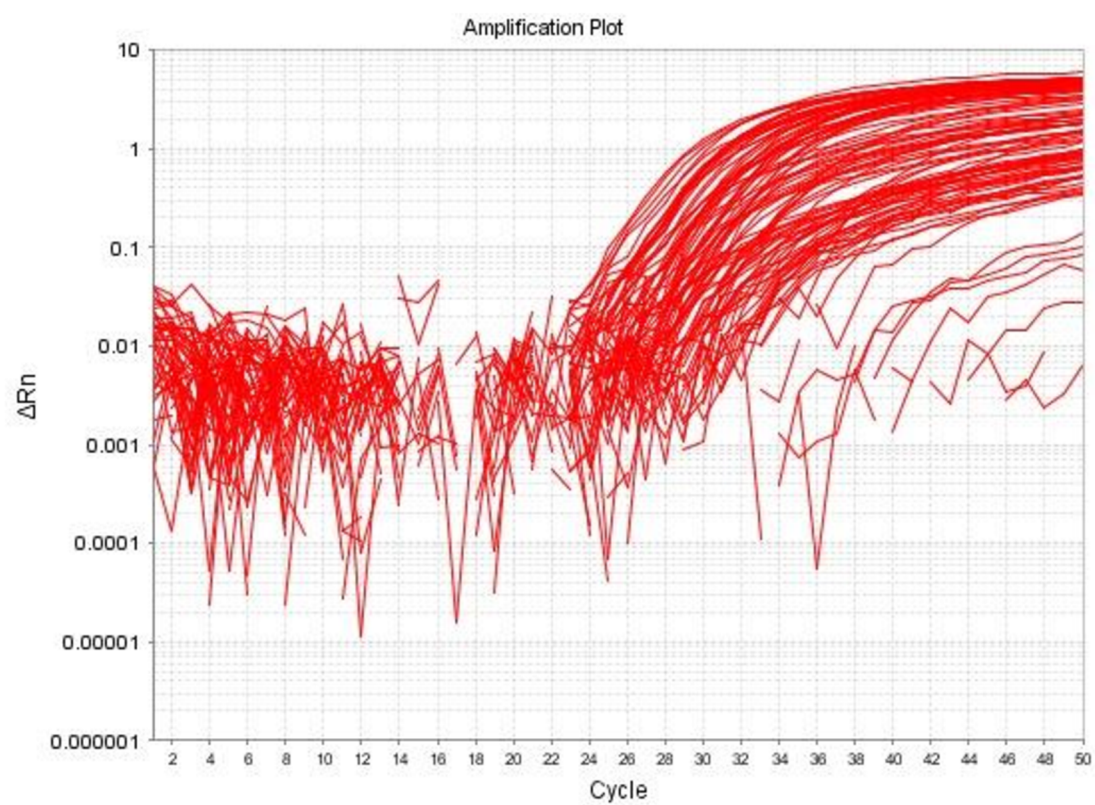

Legend

rs1786814

Supplement: Supplementary file 2 — Supplementary information [file 41390_2024_3400_MOESM2_ESM.pdf]
